# Pneumatic hydrodynamics influence transplastomic protein yields and biological responses during *in vitro* shoot regeneration of *Nicotiana tabacum* callus: Implications for bioprocess routes to plant-made biopharmaceuticals

**DOI:** 10.1016/j.bej.2016.10.007

**Published:** 2017-01-15

**Authors:** Sherwin S. Barretto, Franck Michoux, Klaus Hellgardt, Peter J. Nixon

**Affiliations:** a Department of Life Sciences, Imperial College London, South Kensington, London SW7 2AZ, United Kingdom; b Department of Chemical Engineering, Imperial College London, South Kensington, London SW7 2AZ, United Kingdom; c Alkion Biopharma SAS, Pépinière Entreprise Genopole, 4 rue Pierre Fontaine, 91058, Evry, France

**Keywords:** CIM, callus induction medium, kDa, kiloDalton, MS medium, Murashige & Skoog medium, RITA**^®^**, recipient for automated temporary immersion (translated from French), SDS-PAGE, sodium dodecyl sulphate polyacrylamide gel electrophoresis, TetC, fragment C of tetanus toxin, TF, triphenylformazan, TIB, temporary immersion bioreactor, TSP, total soluble protein, TTC, 2,3,5-triphenyltetrazolium chloride, Transplastomic protein, Biopharmaceutical, Temporary immersion culture, *in vitro* organogenesis, Hydrodynamics, Pneumatic energy dissipation

## Abstract

Transplastomic plants are capable of high-yield production of recombinant biopharmaceutical proteins. Plant tissue culture combines advantages of agricultural cultivation with the bioprocess consistency associated with suspension culture. Overexpression of recombinant proteins through regeneration of transplastomic *Nicotiana tabacum* shoots from callus tissue in RITA^®^ temporary immersion bioreactors has been previously demonstrated. In this study we investigated the hydrodynamics of periodic pneumatic suspension of liquid medium during temporary immersion culture (4 min aeration every 8 h), and the impact on biological responses and transplastomic expression of fragment C of tetanus toxin (TetC). Biomass was grown under a range of aeration rates for 3, 20 and 40-day durations. Growth, mitochondrial activity (a viability indicator) and TetC protein yields were correlated against the hydrodynamic parameters, shear rate and energy dissipation rate (per kg of medium). A critical aeration rate of 440 ml min^−1^ was identified, corresponding to a shear rate of 96.7 s^−1^, pneumatic power input of 8.8 mW kg^−1^ and initial 20-day pneumatic energy dissipation of 127 J kg^−1^, at which significant reductions in biomass accumulation and mitochondrial activity were observed. There was an exponential decline in TetC yields with increasing aeration rates at 40 days, across the entire range of conditions tested. These observations have important implications for the optimisation and scale-up of transplastomic plant tissue culture bioprocesses for biopharmaceutical production.

## Highlights

• Tissue culture is advantageous for ‘molecular farming’ in transplastomic plants.
• *In vitro* shoot regeneration of *N. tabacum* callus was carried out.
• Biological responses were correlated to pneumatic hydrodynamic parameters.
• Reduced growth and mitochondrial activity occurred above a critical shear rate.
• Transplastomic protein yield declined exponentially with increasing aeration.

## 1 Introduction

The use of plants for the heterologous expression of biopharmaceutical proteins, referred to as ‘molecular farming’, is emerging as an alternative to established microbial and mammalian hosts in recent decades [1], [2], [3], [4]. There are several benefits of molecular farming. Plants are capable of the expression of a wide range of mammalian proteins, including monoclonal antibodies, therapeutic enzymes, blood proteins, cytokines, growth factors and hormones [4]. Similar to mammalian cells, plants can perform post-translational modifications required for protein functional activity, including folding, disulphide bond formation, subunit assembly, proteolysis and glycosylation [4]. Hence plant cultures may be considered comparable to conventional mammalian cell cultures, such as CHO cells [4]. Plants do not harbour human pathogens and, unlike bacteria, do not produce endotoxins [5]. Hence, protein production in plants may provide additional safety margins and fewer regulatory hurdles, compared to mammalian and microbial hosts.

Within the field of molecular farming, a number of genetic and cellular engineering approaches have been attempted to enhance product yields and aid bioprocessing of plant-made proteins. Chloroplasts, the site of oxygenic photosynthesis in plants, belong to a class of organelles known as plastids. Plastids possess their own genome, a double stranded circular DNA molecule of 130–160 kb containing 120–130 genes [6]. ‘Transplastomics’ refers to the genetic transformation of the plastid genome, usually for heterologous expression and localisation of foreign proteins within chloroplasts. The main advantage of protein expression in transplastomic plants is the demonstrated high capacity of chloroplasts to overexpress and accumulate foreign proteins, giving high product yields [7]. This is due to polyploidy with approximately 100 chloroplasts per leaf cell containing a total of 10,000 copies of plastid DNA [8]. Plastids are maternally inherited, though low-level leakages of transgenes in pollen may occur [9]. Transplastomic plants do not offer absolute safety against ‘gene pollution’, although if grown in *in vitro* cell suspension, tissue culture or fully-contained greenhouses, can be part of an integrated biosafety strategy [10].

Historically, research efforts into the establishment and clinical development of plant-made biopharmaceuticals have focussed on agricultural cultivation of soil-based plants, perhaps due to relatively high protein yields, straightforward cultivation and low upfront investment [11], [2]. However, for large-scale industrial production of plant-made biopharmaceuticals, bioprocessing routes based on *in vitro* biomass growth in bioreactors are being advocated [12]. The advantages of *in vitro* approaches include GMP (‘good manufacturing practice’) production, reduced variability in product yields compared to soil propagation, greater control and standardisation of process conditions and improved biosafety through fully-contained biomass growth. There is over 50 years of innovation in the bioprocessing of plant cell suspension cultures, historically for the synthesis of active compounds (secondary metabolites), though these are now being adopted for expression of protein biopharmaceuticals [13], [4], [14]. Despite decades of bioprocess optimisation, low recombinant protein yields are still a major limitation of cell suspension culture [15]. Recently, *in vitro* culture of differentiated shoots is being investigated for recombinant protein production, and is particularly suited to transplastomic protein expression in photosynthetically-active chloroplasts [16]. *In vitro* plant tissue culture is a potential alternative to both agricultural propagation and cell suspension, combining the advantages of whole tissue cultivation with the ease of manipulating bioprocess conditions associated with suspension culture in bioreactors [12].

Similar to conventional microbial bioreactors, bioreactors for plant cell and tissue culture have been based on stirred tank, bubble column and airlift reactor configurations [17], [12]. An alternative to these is the ‘temporary immersion’- type culture system. In temporary immersion bioreactors (TIBs), biomass is not permanently submerged, but periodically immersed and drained in liquid media. Avoidance of permanent submergence facilitates adequate oxygen transfer, reduces physiological stress and encourages plant growth and differentiation [18]. TIBs have been used for experimental and commercial micropropagation for the high multiplication generation of high-quality shoots and plantlets of several species [18] and more recently for expression of recombinant proteins [16], [19], [20].

Liquid cultures require adequate mixing for effective nutrient and oxygen transfer and suspension homogeneity. Mixing occurs through mechanical agitation and sparging of air in stirred tank bioreactors, and sparging alone in bubble column and airlift reactors. Hydrodynamic shear stresses are required for adequate mixing, associated with velocity gradients within the agitated or sparged liquid [21]. However, numerous studies involving microbial, mammalian and plant cell cultures have demonstrated that hydrodynamic shear can adversely affect growth, metabolism and product formation [22]. Plant cells are particularly sensitive to shear damage because of their relatively large size compared to microbes, rigid cell wall and large vacuoles [23]. It is therefore important to characterise the shear environment of culture systems for the growth of plant biomass, to reduce cellular damage and loss of product yield due to hydrodynamics. Mixing may not be very important in TIBs as contact between biomass and liquid medium is periodic and nutrient transfer occurs via a thin film of medium on the surface of the tissues between immersions. However, hydrodynamic shear during periodic immersion can affect biological responses, and prior to this study, this has not been systematically investigated in detail.

The use of the RITA^®^ temporary immersion bioreactor system for fully-contained recombinant protein expression in transplastomic tobacco (*Nicotiana tabacum* cv. Petite Havana), through regeneration of shoots from callus tissue (*in vitro* organogenesis), has been previously pursued by our group [16], [19], [20]. The study outlined in this publication is in continuity with these pioneering studies. In RITA^®^ bioreactors, periodic suspension of liquid media is the mode of nutrient transfer to biomass. This occurs through pneumatic power input provided by the isothermal expansion of sparged air [24]. In this respect, the RITA^®^ system is comparable to bubble column bioreactors. The aim of this study is to correlate *N. tabacum* growth, mitochondrial activity and accumulation of plastid-encoded recombinant protein, TetC, with the hydrodynamics of pneumatic immersion. TetC is a 47 kDa C-terminal fragment (‘fragment C’) of tetanus toxin that can be used as a subunit vaccine against tetanus [25]. Transformation and expression of TetC in tobacco chloroplasts has been previously described [16], [20], [26], [27].

## 2 Materials and methods

### 2.1 Overview of experimental workflow

Temporary immersion cultures were undertaken at various air flow rates during pneumatic immersion. Differential air flow rates of 38, 45, 165, 376 and 440 ml min^−1^ were used. A number of hydrodynamic parameters were derived from air flow rate; these are shear rate, energy dissipation rate, and total energy dissipation over a 20-day duration (Table 1). Cultures were undertaken as duplicates and biomass was harvested after 3, 20 or 40-day durations. The estimated shear rates and power dissipation rates are the initial rates at the start of the culture period, because callus aggregates are most susceptible to shear damage just after inoculation, and before formation of functional organs. Since each TIB is inoculated with small amount of callus aggregates, the impact of inocula on system properties is assumed to be negligible and the system is initially considered a two-phase system. After the allotted culture durations, biomass was harvested and weighed. Samples were taken for analysis of mitochondrial dehydrogenase activity (as a measure of biomass viability) and TetC expression. SDS-PAGE and immunoblot analysis was undertaken to assess TetC expression. Biomass accumulation, mitochondrial activity and TetC yield were correlated against the hydrodynamic parameters, in order to understand the biological responses of biomass to shear forces generated during periodic pneumatic suspension of the medium.

### 2.2 Generation of inoculum and temporary immersion culture

The transplastomic *Nicotiana tabacum* cv. Petit Havana line Nt-pJST12, expressing TetC, was used as a model system in the described study. The transformation and generation of this line has been described previously [26], [27]. A detailed outline of the procedures we used for plant tissue culture and recombinant protein expression analysis has been described previously [16]. All plant growth was undertaken at 25 °C, under a 16 h photoperiod at an approximate light intensity of 45–120 μmol photons m^−2^ s^−1^. At all stages, tissue culture media based on Murashige & Skoog (MS) basal medium [28] and 3% (w/v) sucrose (30 g l^−1^) were used, set at pH 5.8. All medium components were provided by Sigma (St Louis, MO, USA) unless otherwise stated. For all solidified media, 8 g l^−1^ agar (gelling agent) was added. Aseptic practice was adopted, involving the autoclaving of all media and vessels at 120 °C (103 kPa) for 20 min and the use of laminar air flow hoods. To exclude microbial contamination, all media were supplemented with 500 mgl^−1^ spectinomycin antibiotic (added after autoclaving) and 1 ml l^−1^ Plant Preservative Mixture™ biocide (Plant Cell Technology, Washington DC, USA). The use of spectinomycin as an anti-bacterial agent is a vestige of its earlier application as a selection pressure for enriching recombinant plastid DNA and obtaining homoplastomy after transformation. The transformation cassette includes the *aadA* gene as a selectable marker, which encodes aminoglycoside 3″-adenylyl transferase, conferring spectonomycin resistance [7].

Sterilised seeds were germinated *in vitro* on solidified MS medium in Magenta vessels. Small sections of leaves from germinated seedlings were plated onto callus induction medium (CIM), which is solidified MS medium supplemented with 1 mg l^−1^ 1-napthaleneacetic acid (NAA), 0.1 mg l^−1^ kinetin (K) and agar, for formation of callus tissue. Fine callus suspensions were cultured by loading 30 g l^−1^ friable callus in Erlenmeyer flasks with liquid CIM orbital shaken at 140 rpm and growth to exponential phase. These shaken suspension cultures produced fine uniform callus aggregates, as suitable inocula for temporary immersion culture.

**Fig. 1:**
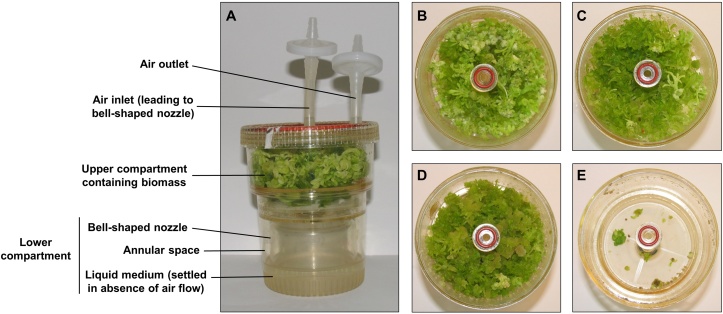
(a) RITA^®^ temporary immersion bioreactor. During periodic pneumatic immersion, air inflow and isothermal expansion in the nozzle and annulus of the lower compartment (containing liquid medium) results in gas hold-up in the lower compartment, causing displacement and suspension of the medium in the upper biomass-containing compartment. When air flow stops, the liquid medium settles to the lower compartment under gravity. Visual demonstration of reduced physiological health of 40-day old biomass with increasing air flow rate, at (b) 38 ml min^−1^, (c) 45 ml min^−1^, (d) 376 ml min^−1^ and (e) 440 ml min^−1^.

Shoot regeneration from callus tissue was undertaken in 0.5 l RITA^®^ temporary immersion bioreactors (Vitropic, CIRAD, France), using shoot regeneration medium, based on MS medium supplemented with 0.1 μM thidiazuron (TDZ). The RITA^®^ temporary immersion system consists of an upper compartment containing biomass and lower compartment containing liquid medium (Fig. 1(a)). This lower compartment is composed of a bell-shaped nozzle and annular space. The key dimensions of the biomass compartment of the cylindrical RITA^®^ bioreactor are as follows: height, 6.60 cm; volume, 538 cm^2^; average cross-sectional area of biomass compartment, 81.51 cm^2^; average hydraulic diameter of biomass compartment, 10.19 cm. The biomass compartment comprises of the annular space between the outer circumference and the central ‘stem’ air inlet pipe; for accuracy, the above dimensions for volume and cross-sectional area do not include this inlet pipe. 0.5 g of cell aggregates, were loaded into each bioreactor. Immersion of biomass in the medium was achieved through periodic sparging of air from an air pump and manifold via silicone tubing, at a duration and frequency of 4 mins every 8 h. Neither the medium nor the biomass suspended in the aerated medium produce foam, due to the lack of foaming agents either in the medium composition or secreted by biomass. Cultures were grown under various air flow rates during pneumatic immersion. The air flow rate was controlled with air control valves in the manifold. After the allotted culture duration (3, 20 or 40 days) the biomass was harvested and samples were taken for viability assays and analysis of transplastomic protein accumulation. Harvested biomass was weighed for ‘fresh biomass’ determination, and then dried at 80 °C for 48 h prior to weighing for ‘dry biomass’ determination.

### 2.3 Viability assay of biomass

This viability assay is based on the reduction of 2,3,5-triphenyltetrazolium chloride (TTC) to insoluble red triphenylformazan (TF) by mitochondrial dehydrogenase activity, as an indicator of plant cell viability [29]. 400 mg of fresh biomass were incubated in 7.5 ml of 0.1 M sodium phosphate buffer (pH 7.0) with 0.6% (w/v) TTC and 0.05% Tween-20, for 20 h at 30 °C in darkness [30]. After incubation, the triphenylformazan was extracted by decanting the buffer, grinding biomass in liquid nitrogen, transferring the powder to eppendorf tubes, addition of 1.7 ml 96% ethanol, and centrifugation at 10,000*g* for 2 min. The supernatants containing TF were collected and the absorbance at 520 nm was determined.

### 2.4 Total soluble protein extraction, SDS-PAGE and immunoblot analysis

Total soluble protein (TSP) extraction was performed according to the method used by Kanamoto and co-workers [31]. Plant biomass was ground into a fine powder with liquid nitrogen and mixed with protein extraction buffer (PEB) (50 mM HEPES-KOH (pH 7.5), 2 mM DTT, 1 mM EDTA, 10 mM potassium acetate, 5 mM magnesium acetate, and 1 tablet of cOmplete Mini protease inhibitors EDTA-free cocktail (Roche Applied Sciences, Germany) per 5 ml buffer) in a ratio of approximately 100 mg biomass to 100 μl, vortex mixed for 30 s, centrifuged at 18,000*g* for 30 min and the supernatant was collected. The supernatant was centrifuged again to remove any residual pellet, and stored at −80 °C before further analysis. The amount of TSP extractable per unit fresh biomass depends on extraction conditions and must be empirically determined for a new protocol.

The protein concentration in TSP samples was determined using the Bradford Assay (Sigma, St Louis, MO, USA) using bovine serum albumin as a standard [32]. Soluble proteins were resolved by sodium dodecyl sulphate polyacrylamide gel electrophoresis (SDS-PAGE) in 12% polyacrylamide gels using the Bio-Rad mini-gel electrophoresis system (Bio Rad Laboratories, USA). Prior to loading onto gels, TSP samples were solubilised in 4 × solubilisation buffer (consisting of 250 mM Tris-HCl (pH 6.9), 8% (w/v) SDS, 40% (w/v) glycerol, 0.1% (w/v) bromophenol blue and 10% (w/v) β-mercaptoethanol (added freshly for each use)) for 5 mins at 100 °C. 7 μg of protein were loaded into each lane. A recombinant TetC standard (provided by Professor Neil Fairweather, Imperial College London, UK) was loaded as a positive control. Mini-gels were run as duplicates, with one mini-gel stained in Coomassie blue and the other for immunoblot transfer. Following electrophoresis, immunoblot (‘Western blot’) analysis was undertaken. Proteins were transferred from the mini-gel to a 0.2 μm nitrocellulose membrane with the Bio-Rad Mini *trans*-Blot^®^ Electrophoretic Transfer Cell (Bio-Rad Laboratories, USA). Subsequently, TetC detection was performed with anti-TetC antibody (‘primary antibody’) (Professor Neil Fairweather, Imperial College London, UK) diluted 1:3000 and horseradish peroxidase-conjugated goat anti-rabbit IgG diluted 1:10,000 (‘secondary antibody’) (Sigma). Enhanced Chemiluminescence (ECL) was undertaken using the Enhanced Chemiluminescence Detection kit (Amersham Pharmacia, UK). Detection was undertaken using an LAS-3000 CCD digital imaging system (FujiFilm, USA). For comparative analysis of TetC accumulation under various aeration rates, densitometry was conducted using ImageJ software (National Institutes of Health, USA).

## 3 Characterisation of rheological and hydrodynamic parameters

The hydrodynamics of multiphase reactors such as temporary immersion bioreactors depend on the rheological properties of the medium and its density [24]. The tissue culture media, composed of MS media (4.4 g l^−1^) and 3% (w/v) (30 g l^−1^) sucrose, is a Newtonian fluid [33], [34], [35], [36]. For aqueous solutions and suspensions, viscosity is a function of solute concentration [37]. The viscosity (*μ*) of the media was estimated using Mooney’s semi-empirical equation relating viscosity to solute concentration [38]:

$$\mu =\mu_{s}exp\left(\frac{2.5\varphi }{1-\frac{\varphi }{\varphi^{*}}}\right)$$

In this, μ_s_ is the viscosity of the solvent, water, which is 0.000891 Pa s at 25 °C. φ is the volume concentration of solute particles, approaching a critical volume concentration, $\varphi^{*}=0.74$ [39]. The pre-dissolution volume of the non-aqueous components of the medium, MS basal salt mixture (4.4 g l^−1^) and 30 g l^−1^ sucrose was determined to be 35 ml, giving a volume concentration φ of 0.035 (35 ml l^−1^). The viscosity of media is estimated to be 0.000976 Pa s. The density (*ρ*) of the tissue culture media is 1.034 g l^−1^.

In pneumatic bioreactors, the air supply is the only source of power, through isothermal expansion of gas. Two important parameters, the average shear rate and volumetric power input can be determined from the superficial gas velocity (*U*_g_) (m s^−1^) [24]. The superficial gas velocity is simply the volumetric gas flow rate (m^3^ s^−1^) divided by the average cross-sectional area 8.15 × 10^−3^ m^2^.

In pneumatic reactors, the average shear rate (*γ*) (s^−1^) of a Newtonian fluid depends on the superficial gas velocity *U_g_*, as follows the following relationship which is derived from theoretical analysis and consistent with empirical observations [24]:

$$\gamma ={\left[\frac{g\rho U_{g}}{\mu }\right]}^{1/2}$$

The specific pneumatic power input, also known as the rate of energy dissipation $\left(\frac{P_{g}}{V}\right)$ (power per unit volume of medium) (W m^−3^) which applies when the isothermal expansion of gas is the predominant source of power [40], [24], is given by the following equation:

$$\frac{P_{g}}{V}=\rho gU_{g}$$

This can also be expressed in terms of the energy dissipation rate per unit mass of medium (*ε*) (W kg^−1^) by dividing the above equation by the fluid density.

It may be more appropriate to assess cumulative biological responses against cumulative energy dissipation (instead of specific power dissipation) (J kg^−1^) [41]; [42]. This is simply a product of the specific power dissipation (W kg^−1^) and the exposure time (s). In this study the total energy dissipation over the initial 20 days of TI culture is easily calculated, given the immersion duration/frequency of 4 min/8 h. The total exposure time over the initial 20 days is 14,400 s (4 h).

## 4 Results

### 4.1 Effect of shear rate and power dissipation on biomass accumulation

**Fig. 2:**
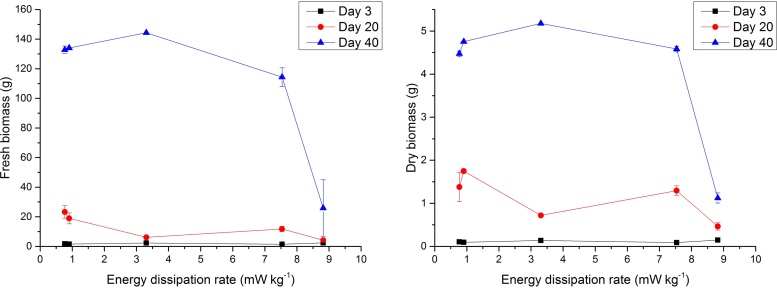
Plots showing the influence of energy dissipation rate on fresh and dry biomass accumulation, after 3, 20 and 40-day cultures. Error bars denote standard errors.

Accumulation of fresh and dry biomass declined with increasing aeration rate for both 20 and 40-day old cultures, corresponding to increasing shear rate and energy dissipation rates (Fig. 2). An increase of air flow rate from 38 to 376 ml min^−1^ (corresponding to a 28.5 to 89.3 s^−1^ change in shear rate, or 0.77–7.53 mW kg^−1^ change in energy dissipation) resulted in 50% and 14% reductions in fresh weight, at 20 and 40 days respectively. However, within this range of flow rates, dry weight was largely unaffected, at both 20 and 40 days. The most significant decrease in growth occurred at 440 ml min^−1^ (equivalent to a shear rate of 96.7 s^−1^ or energy dissipation rate of 8.82 mW kg^−1^). At 20 and 40 days respectively, a 440 ml min^−1^ flow rate resulted in fresh weights of only 4.2 g and 25.9 g per bioreactor, and dry weights of 0.5 g and 1.1 g per bioreactor; this corresponds to fresh weight reductions of 82% and 80% respectively, and dry weight reductions of 66% and 75% respectively, relative to that at 38 ml min^−1^. No significant reductions in biomass growth (either fresh or dry biomass) were observed in 3-day cultures. In 40-day cultures, cell differentiation and shoot formation were observed under all aeration rates tested except at 440 ml min^−1^. A high degree of differentiation was observed in 40-day biomass grown at 38 and 45 ml min^−1^ aeration rates, characterised by clusters with a proliferation of a large number of fully-formed leaves up to 2.5 cm in length. In 40-day old cultures grown at 165 and 376 ml min^−1^, notably less shoot and leaf formation was observed and the biomass was composed of undifferentiated clusters and partially-differentiated clusters with nodular outgrowths that have not fully developed into shoot buds. Organogenesis is time-dependent and shoot formation was observed in 40-day cultures but not 3- or 20-day cultures.

### 4.2 Effect of shear rate and pneumatic power input on mitochondrial activity

**Fig. 3:**
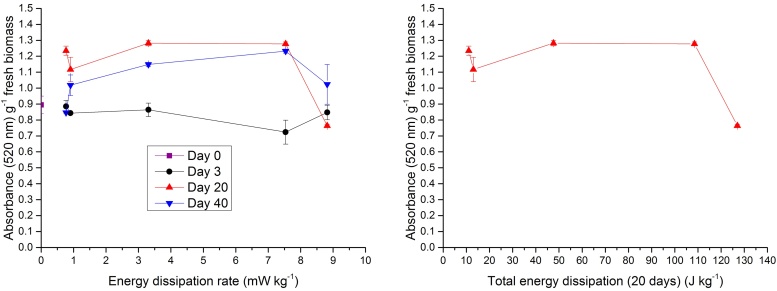
Plots showing the influence of energy dissipation rate after 0, 3, 20 and 40-day cultures and total energy dissipation (after 20 days culture only) on mitochondrial respiratory activity. Error bars denote standard errors.

Dehydrogenase activity of the mitochondrial electron transport chain is an indicator of overall cellular viability and metabolic activity, and can be measured in terms of absorbance (520 nm) of reduced triphenylformazan [30], [29]. Plots showing the effect of hydrodynamic parameters on mitochondrial dehydrogenase activity after 3, 20 and 40-day cultures are shown in Fig. 3. Mitochondrial activity was largely unaffected by air flow rate (and derived parameters, shear rate and energy dissipation rate) at 3 and 20 days between 38 and 376 ml min^−1^, though a shallow increase in mitochondrial activity with increasing hydrodynamics was observed for the 40 days culture. Significant impairment of mitochondrial function is observed at a 440 ml min^−1^, corresponding to an average shear rate of 96.7 s^−1^ and energy dissipation rate of 8.82 mW kg^−1^. At 20 and 40 days, the mitochondrial activity at this aeration rate is 60% and 67% of that at 376 ml min^−1^. This is reflective of the reduction in biomass growth at this flow rate. It is useful to express cell viability as a function of total cumulative energy dissipation [42], which is a product of the energy dissipation rate and exposure time. A plot of mitochondrial activity against total energy dissipation over the first 20 days of temporary immersion culture is presented in Fig. 3. A 17% increase in 20-day total energy dissipation from 108.5 and 127 J kg^−1^ results in a 40% drop in mitochondrial activity.

### 4.3 Effect of shear rate and pneumatic power input on TetC expression

**Fig. 4:**
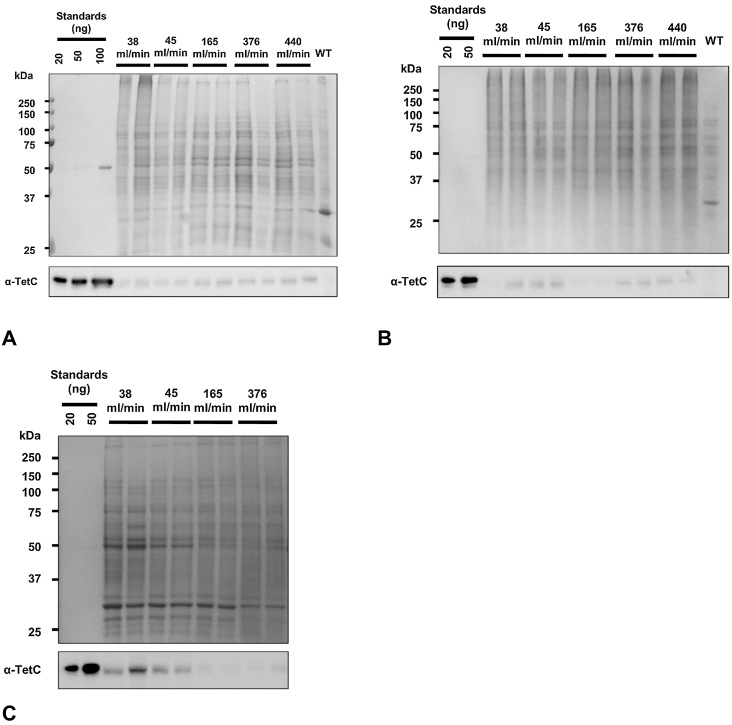
SDS-PAGE and immunoblots showing the effect of air flow rate on TetC expression. (A) 3-day TI culture; (B). 20-day TI culture; (C) 40-day TI culture. 12% acrylamide gel; 7 μg protein loading; Coomassie staining.

SDS-PAGE and immunoblot analysis was undertaken on total soluble protein (TSP) extracted from biomass grown at various air flow rates at 3, 20 and 40-day intervals (Fig. 4). For 3 and 20 days, air flow rate was found to have no discernable effect on TetC expression. However, for the 40-day cultures, TetC expression decreases with increasing air flow rate, as the immunoblot indicates (Fig. 4(c)).

**Fig. 5:**
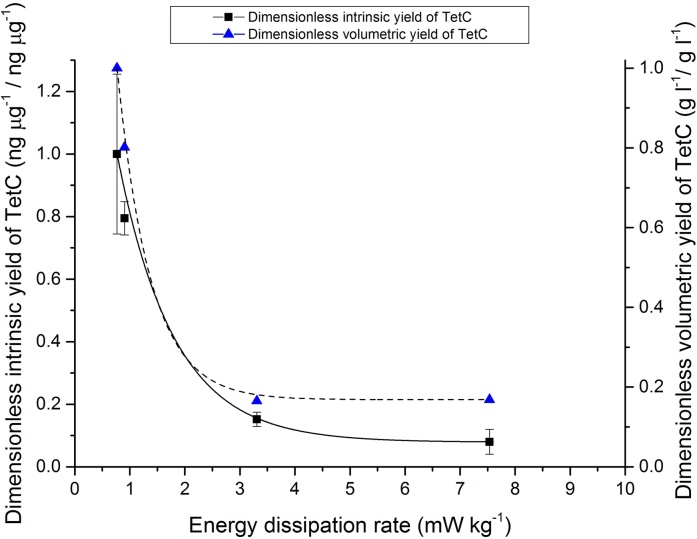
Plot showing the influence of energy dissipation rate on intrinsic and volumetric yields of TetC after 40-day culture. Yields were determined as dimensionless values by dividing by the maximum respective yield, and can thus be placed on a relative scale between 0 and 1. Error bars denote standard errors.

Densitometric analysis was undertaken on the immunoblot shown in Fig. 4(c) to visualise the trend in TetC yield reduction after 40-day culture with parameters derived from air flow rate, shear rate and energy dissipation rate (Fig. 5). The intrinsic yield is the abundance of recombinant protein as a proportion of total soluble protein (TSP), and can be expressed in absolute semi-quantitative terms (% TSP or ng μg^−1^) or relative terms (as fold changes between different conditions). The relative intrinsic yield was determined by immunoblot densitometry and normalisation to total stained protein on a gel [43]. The volumetric yield can be estimated as the product of the intrinsic yield and TSP extractable from a bioreactor culture. The intrinsic yields and estimated volumetric yields of TetC were determined as relative amounts by dividing by the respective maximum yield observed at 38 ml min^−1^ and thus expressed as dimensionless values (on a scale of 0–1). An apparent exponential decay relationship in TetC yield is observed with respect to shear rate and energy dissipation rate. A 4.3-fold increase in air flow rate from 38 ml min^−1^ to 165 ml min^−1^, corresponding to a 2.1-fold increase in shear rate (28.5 s^−1^–59.2 s^−1^) and 4.3-fold increase in energy dissipation rate (0.77 mW kg^−1^–3.31 mW kg^−1^) will lead to a 83% reduction in volumetric yield.

## 5 Discussion

Shear stresses can greatly affect the growth, metabolism and viability of cultured plant cells, aggregates and tissues [23], [44], [12]. The responses of plant cells to hydrodynamic stresses have become a topic of intense systematic investigation in recent years. There are two main approaches to undertaking such investigations, exposing cells to a well-defined hydrodynamic environment (such as a couette, capillary or submerged jet device) for a short duration under non-growth conditions, or exposing cells to shear forces in cultivation vessels under growth conditions [23], [44]. The latter approach was used for this study. Previous studies assessing the effects of shear in plant culture have investigated cell suspension cultures [45], [46], [47], [48], [49], [42], [50], [51], [52]. In contrast, there has been little investigation into the impact of shear on differentiated plant systems [53]. This is the first reported attempt to systematically quantify the impact of hydrodynamics on *in vitro* organogenesis from callus, in the context of transplastomic protein synthesis. Early studies investigated shear effects on production of secondary metabolites in cell suspensions [54], although with the advent of commercial ‘molecular farming’, it is likely that more studies will focus on the impact of hydrodynamics on protein expression yields [45]. When we first used RITA^®^ bioreactors for tobacco shoot regeneration, we observed that high air flows were detrimental to biomass growth. This study represents an attempt to empirically correlate hydrodynamics associated with periodic sparging with cumulative biological responses in terms of biomass growth, metabolic activity and transplastomic protein expression, revealing key implications for industrial bioprocessing and scale-up.

In pneumatic two-phase systems such as TIBs, the hydrodynamics are determined by the air flow rate [24]. Although the fluid dynamics are too complex to fully characterise, two parameters derived from the aeration rate, the average shear rate (γ) and isothermal energy dissipation rate (*ε*) may be correlated against biological responses.

While an increase in aeration from 38 to 376 ml min^−1^ (corresponding to increases in shear rate from 28.5 s^−1^ to 89.3 s^−1^ and energy dissipation from 0.77 mW kg^−1^ to 7.53 mW kg^−1^) did result in a 50% decline in fresh biomass growth at 20 days, there was little impact on dry biomass growth at 3, 20 and 40 days. Similarly, within this range, mitochondrial activity was largely unaffected by increasing shear for 3-day and 20-day cultures, although a steady increase was noted for 40-day cultures. However, the observation of reduced leaf formation with increasing aeration is an indicator of damage to meristems and reduced differentiation potential.

At a critical flow rate of 440 ml min^−1^, corresponding to an average shear rate of 96.7 s^−1^ and energy dissipation rate of 8.82 mW kg^−1^, significant cellular damage is observed for both 20 and 40-day cultures, indicated by reduction in biomass accumulation and mitochondrial activity. A number of authors have suggested the need to establish critical values for energy dissipation to avoid, and thus reduce excessive shear damage of biomass [41], [23], [46], [42]. The results of this study indicate a critical total energy dissipation of 127 J kg^−1^. Total energy dissipation is a more reliable critical parameter than energy dissipation rate, as biological responses against shear damage tend to be cumulative over time [42]. Total energy dissipation over a 20-day culture duration corresponds to the ‘lag’ phase of *in vitro* organogenesis, when there is very little biomass increase and virtually no morphogenesis, hence the culture can be approximated to a two-phase system. During this phase, the callus inoculum slowly proliferates just prior to the rapid increase in meristemic shoot formation. Indeed, this early phase is important in determining the later physiological and metabolic status of the biomass.

TetC expression was found to be especially sensitive to shear damage, and exhibits an apparent exponential decrease in yield with increasing shear conditions. Unlike growth and mitochondrial activity, which exhibited a significant decline with a high air flow rate, the reduction in both intrinsic and volumetric yields of TetC was across the entire range of aeration rates tested. These results indicate a significant reduction in volumetric yield above an average shear rate of 31.0 s^−1^. These results demonstrate that low aeration conditions during pneumatic immersion are necessary to maintain high yields of transplastomic protein expression. Transplastomic protein accumulation is correlated to chloroplast development (maturation of proplastids to photosynthetically-active chloroplasts) and increase in chloroplast number during *in vitro* shoot regeneration. The requirement for mature chloroplasts for high-yield transplastomic protein expression necessitates the formation of differentiated leaves and shoots; cell suspension culture (undifferentiated callus aggregates) is unfeasible for this as intrinsic yields are very low. The decline in transplastomic protein expression, indicative of reduced chloroplast development, is possibly related to the observed meristemic damage and reduced differentiation of functional leaves.

Although no comprehensive studies of hydrodynamic effects on *in vitro* organogenesis of callus have been undertaken previously, the results of this study indicate a number of fundamental approaches that can be employed for the design and scale-up of pneumatic immersion cultures. These results indicate that low aeration rates promote growth, differentiation and transplastomic protein expression. These results indicate that to avoid critical physiological damage, it is advisable not to exceed an average shear rate of 89.3 s^−1^, or total energy dissipation over the first 20 days of 108.5 J kg^−1^. However, the observed steady decline in transplastomic protein expression suggests to avoid exceeding an average shear rate of 31.0 s^−1^ to maintain high biopharmaceutical yields. Consideration of these important parameters can be used in bioprocess scale-up for bioreactor design and operation. As the interest in plant tissue cultures for molecular farming grows, there will also be need to elucidate the underlying cellular and physiological mechanisms of shear damage to differentiated plant tissues.

## 6 Conclusion

In recent decades, there has been much progress in transplastomic transformation strategies. To achieve high-yield expression of transplastomic proteins by *in vitro* tissue culture, formation of differentiated shoots is required for development of host chloroplasts. However, there has been little emphasis on the bioprocess optimisation for large-scale culture of differentiated transplastomic plant tissues. The study presented here demonstrates the shear sensitivity of *in vitro* shoot regeneration from *N. tabacum* callus by periodic pneumatic immersion in media. While significant reductions in growth and metabolic activity were observed at a shear rate of 96.7 s^−1^, corresponding to a critical pneumatic energy dissipation for the initial 20 days of 127 J kg^−1^, transplastomic protein expression was found to decline exponentially with increasing shear, even at moderate aeration rates. These results reveal important hydrodynamic parameters that must be taken into account for the design and scale-up of pneumatic bioreactors for plant tissue culture, especially for ‘molecular farming’ of transplastomic biopharmaceuticals. As more plant-made biopharmaceuticals enter into clinical and commercial development, it is likely that such insights will be instrumental in the design and optimisation of robust transplastomic plant-based bioprocess routes.

## Figures and Tables

**Table 1:** Parameters derived from air flow rate.

Air flow rate (*Q*) (ml min^−1^)	Air flow rate (*Q*) (m^3^ s^−1^)	Superficial gas velocity (*U*_g_) (m s^−1^)	Average shear rate (*γ*) (s^−1^)	Energy dissipation rate per unit volume of medium (pneumatic power input) $\left(\frac{P_{g}}{V}\right)$ (W m^−3^)	Energy dissipation rate per unit mass of medium (pneumatic power input) (*ε*) (mW kg^−1^)	Total energy dissipation (per unit mass of medium) after 20 days culture (J kg^−1^)
38.32	6.39 × 10^−7^	7.83 × 10^−5^	28.53	0.79	0.77	11.06
45.11	7.52 × 10^−7^	9.22 × 10^−5^	30.96	0.94	0.90	13.03
165.00	2.75 × 10^−6^	3.37 × 10^−4^	59.20	3.42	3.31	47.64
375.75	6.26 × 10^−6^	7.68 × 10^−4^	89.34	7.79	7.53	108.50
439.81	7.33 × 10^−6^	8.99 × 10^−4^	96.66	9.12	8.82	127.00
